# Marked improvement in severe postherpetic itching following an epidural block: a case report

**DOI:** 10.1186/s40981-023-00618-8

**Published:** 2023-05-15

**Authors:** Shinju Obara, Rieko Oishi, Yuko Nakano, Shin Kurosawa, Satoki Inoue

**Affiliations:** 1 Department of Anesthesiology, Fukushima Medical University, 1 Hikarigaoka, Fukushima, Fukushima 960-1295 Japan; 2 Center for Pain Management, Fukushima Medical University Hospital, 1 Hikarigaoka, Fukushima, Fukushima 960-1295 Japan

To the Editor:

Although postherpetic itching (PHI) is a sequalae of herpes zoster infection, the reports of it reaching a level that threatens the quality of life are limited. Herein, we describe a case in which an epidural block effectively treated a severe case of PHI. A 72-year-old woman developed left buttock pain 37 days before her first visit to our clinic. Two weeks later, she noticed a rash in the same area. A local physician diagnosed her with herpes zoster and initiated amenamevir 400 mg/day for 7 days. Three days later, she was referred to a dermatologist for persistent pain. She was treated with pregabalin 225 mg/day and acetaminophen 2 g/day. Although the pain considerably improved, she developed intense itching and dizziness. Topical capsaicin was ineffective, and the patient was referred to our pain clinic. The skin rash in the left S1 area was already crusted over. Her visual analog scale (VAS) score for itching was 50/100 mm during the day and 100/100 mm at night; her VAS score for pain was 30/100 mm. Her itching caused insomnia. She was diagnosed with herpes zoster-related pain and PHI in the left S1 region. An epidural block (L5/S1 level; combination of 5 ml 1% lidocaine and 1.65 mg dexamethasone) was administered. The itching immediately disappeared, causing only discomfort; this anti-itching effect persisted thereafter. Amitriptyline 10 mg/day was additionally administered, but she took it only once and terminated by herself because of drowsiness and realizing the effect of epidural block. A total of five epidural blocks were administered every 1–2 weeks, and the oral pregabalin and acetaminophen were tapered off. All medications were stopped on the 71st day after the first visit.

In herpes zoster, 17–62% and 30–58% of patients develop itching in the acute and chronic phases, respectively [1, 2]. However, PHI can be underestimated because the patients’ main symptom is usually pain. Cases of itching that severely reduce a patient’s quality of life are rarely reported [3–5].

The skin is innervated with small unmyelinated (C-fiber) and thinly myelinated (A-delta fibers) axons that transmit itch and pain sensation (nociception) [6]. Neuropathic itch occurs when small nerve fibers are damaged or injured; patients with herpes zoster infection often perceive such a sensation after the spontaneous firing of the nerves [6, 7]. In our case, itching occurred despite improvement of the rash and resembled the time course of acute and subacute herpetic neuralgia, leading us to suspect itching due to neuropathy. Oaklander stated that the administration of local anesthetics inhibits neuronal firing and affects small fiber firing, which reduces neuropathic itch [8]. Yamanaka et al. reported a case where the supraorbital nerve block was effective for PHI [5]. Additionally, a calcium channel α2δ ligand is reportedly effective for PHI [4]. The ligand was only effective for pain in our patient, even at the maximum dose; it was ineffective against itching. In our patient, the epidural block eliminated the PHI by blocking the signal input from C-fibers and A-delta fibers. The mechanism by which this effect persisted may be similar to the analgesic effect interrupting the nociceptive stimuli by nerve blockade in the areas of peripheral sensitization; however, the details remain unknown. The anti-inflammatory effects of steroids may also have been effective. The worst complication of PHI is self-injury caused by continued painless scratching because of colocalizing severe sensory loss [8]. Our patient did not complain of decreased sensation, but the epidural block could have indirectly controlled skin damage from scratching. In conclusion, the epidural block, which is commonly administered for pain, may be a possible treatment option for PHI.

## Data Availability

Not applicable.

## Abbreviations

PHI: Postherpetic itching
VAS: Visual analog scale
